# Awake Prone Positioning in Non-Intubated Non-COVID-19 ARDS: A Comprehensive Review

**DOI:** 10.3390/arm94040053

**Published:** 2026-07-27

**Authors:** Mairi Ziaka, Aristomenis Exadaktylos

**Affiliations:** 1 Department of Emergency Medicine, Inselspital, University Hospital, University of Bern, 3010 Bern, Switzerland; 2 Department of Internal Emergency Medicine, Thusis Hospital, 7430 Thusis, Switzerland; 3 Academic Department of Emergency Medicine, School of Medicine, University of Cyprus, 1678 Nicosia, Cyprus

**Keywords:** acute hypoxemic respiratory failure, ARDS, awake prone positioning, intubation, mechanical ventilation, patient self-inflicted lung injury

## Abstract

**Highlights:**

**What are the main findings?**

- Evidence supporting APP in non-COVID-19 AHRF/ARDS remains scarce and is mainly derived from small observational studies.
- APP may improve oxygenation through enhanced ventilation distribution and V/Q matching, although its impact on clinical outcomes remains uncertain.

**What are the implications of the main findings?**

- APP appears to be a safe and well-tolerated adjunctive strategy in selected non-intubated patients with AHRF when combined with appropriate respiratory support and close monitoring.
- Future trials should identify patients most likely to benefit and define optimal APP protocols.

**Abstract:**

Despite advances in the understanding of the pathophysiology of acute respiratory distress syndrome (ARDS), treatment options remain limited and are mainly supportive, while mortality remains high. Prone positioning (PP) has been shown to improve oxygenation and lung mechanics in ARDS by reducing the imbalance in ventilation distribution between ventral and dorsal lung regions, altering pulmonary blood flow distribution, modifying the density distribution of edematous lung tissue, and limiting areas with low ventilation–perfusion ratios. During the coronavirus disease 2019 (COVID-19) pandemic, the use of PP, referred to as awake prone positioning (APP), was extended to non-intubated patients with severe hypoxemic respiratory failure. However, several concerns remain, including worsening oxygenation following the transition from prone to supine position, the potential development of patient self-inflicted lung injury (P-SILI), and delays in endotracheal intubation and initiation of invasive mechanical ventilation. Evidence regarding the use of APP in non-COVID-19 ARDS is scarce and consists mainly of small case series and a limited number of prospective studies with small and heterogeneous populations. Therefore, in the present work, we aim to summarize the existing evidence on APP in non-COVID-19 ARDS and acute hypoxemic respiratory failure (AHRF), describe the underlying pathophysiological mechanisms, and highlight areas for future research.

## 1. Introduction

ARDS is characterized by sudden, severe respiratory insufficiency associated with non-cardiogenic pulmonary edema and disrupted alveolocapillary permeability, leading to marked hypoxemia, bilateral pulmonary opacities, and excessive pulmonary inflammatory reactions [1]. ARDS can occur in the context of various pathologies, either of extrapulmonary origin—such as sepsis, pancreatitis, transfusion of blood products, and non-thoracic major trauma—or of pulmonary origin, such as pneumonia, inhalational injury, and aspiration of gastric contents [2,3]. However, almost six decades after its first description, the mortality of ARDS remains persistently high, with limited therapeutic options, which is largely linked to the significant heterogeneity of the syndrome [4].

In recent years, it has been highlighted that non-invasive ventilation (NIV) may be useful in AHRF and mild to moderate ARDS due to its ability to assist ventilation, improve gas exchange, and reduce the work of breathing. Consequently, it may decrease the need for intubation and invasive mechanical ventilation, thereby also reducing associated complications [5,6]. However, major considerations remain such as the delay in intubation, which is associated with higher mortality in patients who experience NIV failure, and the higher tidal volumes (TVs) generated during NIV, which may contribute to additional lung injury (LI) and increased mortality [6]. Following the introduction of high-flow nasal cannula (HFNC), the optimal ventilatory support modality for patients with AHRF became a subject of debate. Indeed, randomized clinical trials comparing facemask NIV with HFNC did not demonstrate superiority of NIV [7,8,9,10,11,12], while two studies reported higher intubation and mortality rates with NIV [7,8].

The value of PP in improving oxygenation and consequently the survival rate in patients with severe LI and ARDS has been well established for more than 50 years [13]. Physiological studies highlight the impact of various mechanisms on gas exchange, which partly depend on the disease state and individual patient characteristics. These mechanisms include minimizing the imbalance in ventilation distribution between ventral and dorsal lung regions, altering blood flow distribution, modifying the density distribution of edematous lung tissue, and limiting areas with low ventilation–perfusion ratios [14,15,16,17].

While PP is one of the evidence-based measures in the management of intubated patients with ARDS, its use in non-intubated patients with severe LI, referred to as APP, emerged during the COVID-19 pandemic, when it was frequently used as a rescue therapy for non-intubated COVID-19 patients [18,19]. Indeed, despite the controversial results of observational studies, randomized controlled trials, and meta-analyses regarding the impact of APP on improving oxygenation through increased alveolar recruitment in collapsed lung regions and enhanced functional residual capacity and its ability to avoid intubation [18,20], APP was established early as a standard of care for patients with severe COVID-19. However, despite its wide acceptance, uncertainties exist regarding its contribution to the development of P-SILI due to delays in initiating mechanical ventilation [21].

The existing data on the use of APP in non-COVID-19 ARDS are scarce, consisting mainly of small case series and a limited number of small prospective observational studies. Most reports describe avoidance of intubation in more than 50% of patients and significant improvement in oxygenation. Importantly, in all included patients, APP was combined with NIV and/or HFNC [5,22,23,24,25].

Given the scarcity of evidence regarding PP in non-intubated, non-COVID-19 patients, we provide a comprehensive literature review about APP in AHRF and ARDS of non-COVID-19 origin, discuss existing evidence and pathophysiological insights, and outline issues for future research. Since AHRF in early COVID-19 is primarily driven by bronchial shunting and perfusion disturbances rather than alveolar collapse, given that the lung tissue is almost normally inflated, the beneficial effects of PP are attributed mainly to improvements in ventilation–perfusion (V/Q) mismatch, predominantly influenced by perfusion characteristics [26]. Therefore, the present work will not address APP in COVID-19 patients.

## 2. Methodology

A comprehensive literature search was conducted using PubMed to identify relevant studies investigating prone positioning in awake, non-intubated patients with AHRF and/or ARDS. Search terms included “acute respiratory distress syndrome,” “lung injury,” “hypoxemic respiratory failure,” “prone positioning,” “awake prone positioning,” “awake pronation,” “mechanical ventilation,” “non-invasive ventilation,” “high-flow nasal cannula,” “patient self-inflicted lung injury,” “haemodynamics,” and “critical care.” Boolean operators (AND, OR) and truncation were used to refine and optimize the search strategy. The search was restricted to articles published in English over the past 20 years (2005–2026), although key earlier publications were incorporated to provide a historical perspective and foundational knowledge.

Studies published up to June 2026 concerning APP in non-COVID-19 AHRF and/or ARDS were considered eligible for inclusion in this narrative review. Both peer-reviewed articles and preprints were included. Publications involving patients with COVID-19-related AHRF, editorials, conference abstracts without full-text availability, and articles written in languages other than English were excluded.

Following the removal of duplicates and initial screening of abstracts, two authors (MZ and AE) independently reviewed the full texts of potentially relevant articles and selected studies based on predefined inclusion and exclusion criteria. In addition, reference lists of eligible studies were manually screened to identify further relevant publications. Original research articles were included and, given the limited availability of evidence in this field, case reports and case series were also considered. For case reports or case series describing multiple patients, data were extracted only from patients fulfilling the inclusion criteria (i.e., APP in non-COVID-19 AHRF and/or ARDS).

Extracted data included study design, patient characteristics, respiratory support modality, duration of APP, and the main physiological and clinical findings. The final literature search was performed on 10 June 2026.

## 3. Physiological Principles of Prone Positioning

### 3.1. Pulmonary Effects

**Figure 1:**
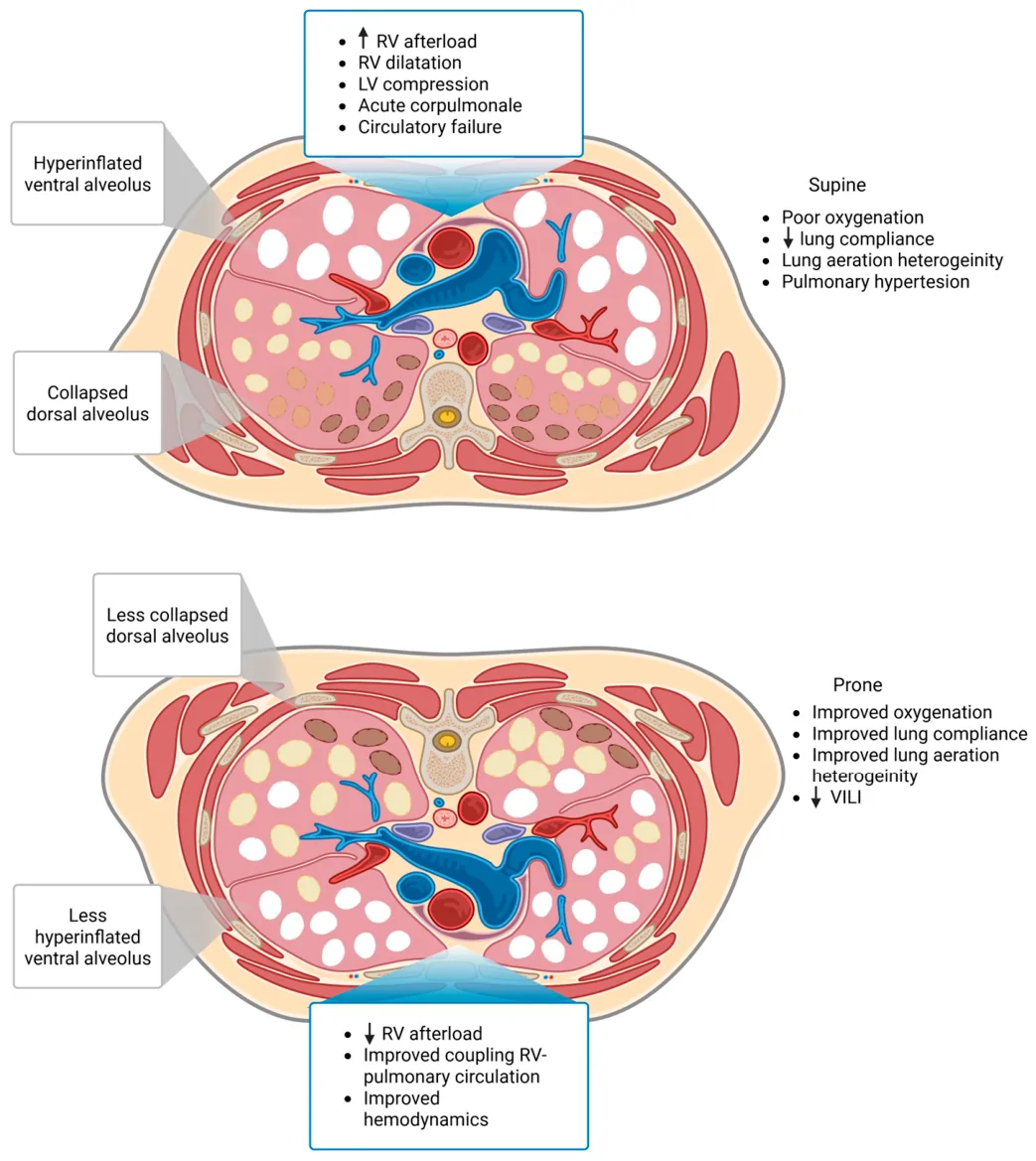
Effects of PP on lung aeration and hemodynamics. PP improves lung aeration and V/Q matching, thereby reducing hypoxic pulmonary vasoconstriction and pulmonary vascular resistance. These physiological changes decrease RV afterload, improve RV–pulmonary circulation coupling, and may lead to improved hemodynamics.

One of the main pathophysiological features of ARDS is vascular hyperpermeability, which leads to increased lung weight and a reduction in well-aerated lung regions due to inflammatory injury of the alveolo-capillary barrier [4,27]. In the supine position, a reduction in well-aerated lung regions, up to complete collapse due to fluid accumulation, occurs along the sternum–vertebral axis (Figure 1). This phenomenon is well explained by the “sponge model,” which conceptualizes the lung as a wet sponge that absorbs fluid and causes gas to shift from dependent to less-dependent regions [28], a pathophysiological understanding that led Professor Gattinoni in the mid-1980s to describe the “baby lung” concept [29]. The “baby lung” concept refers to a functional rather than anatomical model, in which CT imaging studies have highlighted that functional lung volume in patients with ARDS is severely impaired [30,31]. When the patient is positioned from supine to prone, gas shifts from ventral to dorsal lung regions, leading to a reversal of the inflation gradient and redistribution of fluid from dorsal to ventral areas (Figure 1). This results in improved oxygenation, redistribution of transpulmonary forces, and better outcomes, including reduced mortality [28,30].

The inhomogeneity of aerated lung regions in the supine position can also be attributed to the anatomical shape of the lung, which resembles a cone with the dorsal regions forming its base and the chest cavity acting as a cylinder. In the supine position, gravity and negative pleural pressure (P_PL_), which promote close apposition of the chest wall and lung, have differential effects on dorsal and ventral regions. The dorsal areas, compressed by the overlying lung, tend to collapse under the influence of gravity, while the ventral alveolar units become overdistended due to the combined effects of gas redistribution and negative P_PL_, which also results in increased strain on the ventral regions [15,26,32,33]. In contrast, in the PP, the effects of the previously described mechanisms tend to be attenuated, as the dorsal regions expand while the ventral regions, influenced by gravity, adapt to the shape of the thoracic wall. This leads to a more uniform distribution of lung volumes between dependent and non-dependent areas [26,32,34]. Moreover, because P_PL_ depends on a gravity-associated gradient, a stepwise decrease in transpulmonary pressure, the main determinant of lung distension, is observed from non-dependent to dependent regions. As a result, in the supine position, the non-dependent lung regions exhibit higher inflation compared with the dependent regions. This phenomenon is significantly attenuated, and may be largely eliminated, in the PP, where P_PL_ and transpulmonary pressure gradients are reduced, resulting in a more homogeneous distribution of lung inflation and ventilation [26,35,36]. Furthermore, the reduction in the dorsal–ventral P_PL_ gradient during the prone position reduces ventral hyperinflation and dorsal atelectasis, since higher ventral pleural pressure is associated with greater alveolar inflation, whereas lower dorsal P_PL_ promotes alveolar collapse [37].

The diaphragmatic transmission of hydrostatic intra-abdominal pressure to the dependent dorsal lung units in the supine position, together with the weight of the heart, also results in mechanical loading of the dorsal regions, leading to a decrease in functional residual capacity [14,38,39]. The impact of intra-abdominal pressure on the dorsal lung regions is alleviated in the PP, resulting in increased functional residual capacity and more homogeneous alveolar inflation and lung perfusion, with a subsequent improvement in the V/Q ratio [40].

### 3.2. Hemodynamic Effects

It has been convincingly demonstrated that lung recruitment, reductions in pulmonary vasoconstriction, and increases in venous return occurring after PP lead to potential alterations in cardiac output, increased right ventricular preload, and decreased right ventricular (RV) afterload (Figure 1) [41,42,43,44].

Severe acute RV failure may occur in ARDS as a consequence of increased RV afterload due to elevated pulmonary vascular resistance (PVR) and is associated with a worse prognosis [42,45]. The etiology of increased PVR is multifactorial and relates to the principal pathophysiological mechanisms of ARDS, including pulmonary vasoconstriction, pulmonary microthrombus formation, inflammatory cascades, and ventilation-induced hemodynamic alterations. Together, these processes increase RV strain and may lead to RV dilation, impaired systolic and diastolic function, and potentially severe RV dysfunction [46,47,48,49]. By recruiting the pulmonary microvasculature and previously collapsed lung regions, and by attenuating hypoxic pulmonary vasoconstriction through improved arterial oxygenation, PP reduces PVR and RV afterload [42]. Furthermore, PP is associated with reductions in plateau pressure and driving pressure, both of which significantly contribute to RV overload and dysfunction, thereby leading to improved RV function and hemodynamics [50]. In addition, improved oxygenation in the prone position may allow for reductions in positive end-expiratory pressure (PEEP), an important determinant of RV dilation, RV dysfunction, acute cor pulmonale, and circulatory failure, further contributing to improved hemodynamics [51,52].

The effects of PP on cardiac output vary among studies, with some reporting increases [42,53,54,55,56], others decreases [57], and others no significant change [58,59]. It has been shown that the cardiac output response to PP depends on volume status, as PP increases cardiac preload and decreases RV afterload, resulting in increased cardiac output in fluid responders and euvolemic patients [42,53,60]. In a study of 22 patients with mild to severe ARDS, the increase in cardiac output after PP was determined by the gradient between mean systemic pressure and central venous pressure, fluid responsiveness, and changes in venous return resistance. An increase in cardiac index was observed only in fluid responders when the rise in the mean systemic pressure–central venous pressure gradient exceeded the increase in venous return resistance [60]. Besides volume status, elevation of intra-abdominal pressure induced by PP may decrease venous return and subsequently cardiac output, particularly when intra-abdominal pressure exceeds the intramural pressure of the inferior vena cava [60,61,62,63]. However, increases in intra-abdominal pressure that are not sufficient to cause collapse of the inferior vena cava may increase venous return and, consequently, cardiac preload [62,63,64,65].

Regarding left ventricular (LV) function, PP enhances preload and cardiac filling and thus improves cardiac index through attenuation of interventricular septal distortion associated with right ventricular unloading, especially in cor pulmonale [42,66]. In patients with an enlarged RV prior to PP, the reduction in RV–LV interdependence leads to increased LV end-diastolic volume and, consequently, enhanced LV preload [42,66]. Additionally, PP may increase LV afterload due to elevated IAP. However, in patients whose hemodynamic compromise is related to sepsis rather than RV overload, in the presence of hypovolemia, in deeply sedated patients, or in those with very high plateau pressures or receiving high levels of PEEP, prone positioning may result in negative hemodynamic effects [67].

## 4. Impact of Prone Positioning on VILI

Mechanical ventilation is a cornerstone of the therapeutic management of patients with ARDS and severe LI; however, its use may induce significant mechanical alterations, including increased transpulmonary pressure, alveolar overdistension, and repetitive cyclic opening and closure of alveoli. These mechanisms can aggravate LI, worsen hypoxemia, and contribute to ventilator-induced lung injury (VILI), which is associated with increased mortality [1,4]. Among lung-protective mechanical ventilation strategies, including low TV ventilation and the application of PEEP, PP may further reduce VILI by decreasing cyclic alveolar opening and closure and attenuating alveolar overdistension [68,69,70,71,72,73]. Indeed, clinical studies in mechanically ventilated patients with ARDS have shown that PP is associated with a reduction in nonaerated lung areas and tidal hyperinflation at high levels of PEEP, while the combined use of PP and high PEEP results in decreased cyclic recruitment–derecruitment [71]. By increasing end-expiratory transpulmonary pressure, the application of PEEP has the potential to limit atelectasis; however, the optimal titration of PEEP remains controversial, particularly in the presence of distinct ARDS subphenotypes, with recent research supporting an individualized approach based on lung recruitability [74]. Moreover, research indicates that PP may reduce mechanical power, that is, the energy delivered by the ventilator to the respiratory system over time, which is a well-established contributor to VILI [75,76]. Clinical studies demonstrate that PP is associated with increased transpulmonary pressure when PEEP is titrated based on the ARDS Network lower PEEP table, and that this approach mitigates key triggers of VILI, such as mechanical power and transpulmonary driving pressure [77]. Recently, a prospective physiological study in patients with moderate to severe ARDS showed that, during PP, similar transpulmonary pressures and end-expiratory lung volumes are observed at lower airway pressures, and that this is associated with improved oxygenation, increased oxygen delivery, and enhanced cardiac output [76]. The lung-protective effects of PP are further supported by experimental studies demonstrating that PP mitigates VILI by promoting a more homogeneous distribution of lung strain [78].

## 5. Impact of Prone Position on P-SILI

P-SILI refers to the injurious consequence of excessive respiratory drive and increased work of breathing, leading to high TV and elevated transpulmonary pressures in patients with acute respiratory failure, which can aggravate lung damage and are associated with a significantly worse prognosis [79,80,81]. In addition to mechanically ventilated patients with acute respiratory failure, P-SILI can also be observed in spontaneously breathing, non-intubated patients with severe LI or ARDS, who develop patterns of lung damage that resemble VILI [80]. The increased respiratory effort generated by the respiratory musculature can exert deleterious local effects, potentially exacerbating pre-existing lung damage, while elevated transmural pulmonary pressures further contribute to pulmonary vascular leakage, leading to negative pressure pulmonary edema [80]. In addition, the pendelluft phenomenon, defined as intrapulmonary air displacement between different lung regions, occurs during spontaneous breathing in association with negative pleural pressure swings [82,83].

Experimental studies in models of severe ARDS indicate that PP attenuates the effects of respiratory effort during spontaneous breathing by reducing lung stress and regional overinflation, thereby contributing to a reduction in effort-related lung damage [84]. Similarly, clinical studies in patients with severe AHRF and ARDS demonstrate that PP in spontaneously breathing patients is associated with improved gas exchange, reduced respiratory rate, decreased inspiratory effort, and lower dynamic lung stress, potentially preventing or attenuating effort-dependent LI [85,86]. Another physiological mechanism underlying the beneficial effects of PP in reducing P-SILI may involve a form of sensory modulation, whereby improved oxygenation limits respiratory effort, given that hypoxemia is well known to augment respiratory drive through both chemoreceptor stimulation and perceptual pathways [81].

## 6. Existing Data on APP in Non-COVID-19 Respiratory Failure

As mentioned, APP is typically reported as an adjunctive therapy in patients with severe AHRF and mild to moderate ARDS [23,87,88]. Indeed, early use of NIV and HFNC is a common therapeutic strategy in patients with acute AHRF and mild ARDS to reduce the need for endotracheal intubation and invasive mechanical ventilation [6,89,90,91,92]. Although NIV has been shown to achieve greater reductions in esophageal pressure (P_ES_) compared with HFNC [93,94], observational studies indicate that HFNC is often preferred as the initial respiratory support modality, as it is associated with lower transpulmonary pressures and a reduced risk of VILI compared with NIV [95,96]. The FLORALI study, a landmark trial comparing HFNC, standard oxygen therapy, and NIV in patients with non-hypercapnic AHRF, demonstrated significantly lower 90-day mortality and more ventilator-free days at day 28 with HFNC, despite similar intubation rates across groups [7]. Similarly, subsequent clinical studies have failed to demonstrate a clear benefit of NIV in patients with acute non-hypercapnic hypoxemic respiratory failure and mild ARDS [8,9,10]. Therefore, current evidence does not support the routine use of NIV as the initial respiratory support strategy in AHRF when HFNC is available. However, NIV may be evaluated as an alternative when HFNC is unavailable [88].

Traditionally, the PP has been primarily applied in intubated and mechanically ventilated patients. The use of APP became popular during the COVID-19 pandemic as an adjunct to NIV and/or HFNC in patients with severe hypoxemic respiratory failure [97,98,99,100].

More recently, given the beneficial effects of PP on oxygenation, reduction in the need for invasive mechanical ventilation, and improved clinical outcomes, APP has been extended to spontaneously breathing, non-intubated patients with non-COVID-19 AHRF; however, existing data remain limited and consist mainly of case reports, case series, and small, prospective studies [23,101]. The first reported case of PP in awake, non-intubated patients dates back to 1977, when Douglas and colleagues described a 13-year-old patient with acute respiratory failure secondary to acute pancreatitis in the context of hereditary spherocytosis, who was successfully managed with PP and oxygen supplementation delivered via a tight-fitting mask [102]. Valter and co-authors (2003) described four cases of severe AHRF managed with PP to avoid mechanical ventilation and reported a prompt improvement in oxygenation, allowing intubation to be avoided in all patients; no significant complications were observed, and the procedure was well tolerated [22]. Case series in spontaneously breathing, non-intubated lung transplant recipients who developed post-transplant complications and refractory respiratory failure demonstrated favorable outcomes when HFNC and/or NIV were combined with PP, thereby preventing intubation and invasive mechanical ventilation, which can be particularly harmful in this patient population [87,101]. A retrospective study including fifteen awake, non-intubated patients with AHRF, mainly due to pneumonia, managed with different respiratory support devices (oxygen mask, HFNC, continuous positive airway pressure (CPAP), NIV) and PP reported a significant improvement in oxygenation during the PP, without hemodynamic alterations or significant complications, and with good tolerance. The median duration of PP was 3 (2–4) h, with the longest session lasting 8 h. Notably, PP did not affect respiratory rate. Endotracheal intubation was required in two patients, while intensive care unit (ICU) mortality was 20% [23]. A prospective observational study enrolled twenty non-intubated patients with moderate to severe ARDS treated with either HFNC or NIV, with or without PP. The study compared the effectiveness of each respiratory support modality alone and in combination with PP, with the primary outcome being the requirement for endotracheal intubation and invasive mechanical ventilation. Nine patients required endotracheal intubation, the majority of whom were receiving NIV and had a PaO_2_/FiO_2_ ratio < 100 mmHg. In the HFNC combined with PP group, successful outcomes were associated with significantly higher PaO_2_/FiO_2_ ratios than treatment failure, while the relative efficacy of the four respiratory support strategies in improving PaO_2_/FiO_2_ followed the order: HFNC < HFNC + PP ≤ NIV < NIV + PP [5]. In a multicenter retrospective series of six patients with noninfectious severe ARDS, Pérez-Nieto et al. [24] reported that the combination of HFNC or NIV with PP allowed avoidance of endotracheal intubation in four patients. Notably, five patients had a PaO_2_/FiO_2_ ratio < 100 mmHg. Among patients who ultimately required intubation, the combined use of NIV or HFNC with PP was initiated after 48 and 72 h, whereas in most patients who did not require intubation, the combined strategy was started earlier in the disease course (<24 h). PP was performed twice daily, with each session lasting 2–3 h, for a total duration of 2 days (Table 1) [24].

In recent years, physiological studies using electrical impedance tomography (EIT) have investigated the effects of PP on regional lung ventilation and perfusion in awake patients with AHRF (Table 1). Wang et al. reported that PP with an average duration of 2 h resulted in a significant increase in the ROX index (SpO_2_/FiO_2_ divided by respiratory rate) in ten patients with moderate to severe ARDS, and was associated with improved ventilation distribution and greater ventilatory homogeneity [105]. In a prospective physiological study including twenty-four patients with severe AHRF, both COVID-19-associated and non-COVID-19-associated, Chao and colleagues (2024) reported a significant increase in PaO_2_/FiO_2_ after 2 h of PP, along with improved V/Q matching. In patients with diffuse or patchy pulmonary opacities, PP resulted in a significant reduction in intrapulmonary shunt, whereas shunt fraction remained stable in those with a focal pattern of lung infiltrates. Moreover, dead space decreased significantly after PP in patients with focal morphology but remained unchanged in patients with diffuse consolidations. The authors further noted that, among different respiratory support modalities, improvements in oxygenation and V/Q distribution with PP were more frequently observed in patients receiving HFNC therapy [106]. Finally, a physiological crossover study in fifteen patients with severe respiratory failure (eight patients with COVID-19 and seven with non-COVID-19 respiratory failure) reported that proning significantly improved oxygenation, reduced respiratory rate, and increased inspiratory effort, while shifting TV distribution toward dorsal lung regions without altering overall TV. Twenty-seven percent of the overall study population (COVID-19 and non-COVID-19) required endotracheal intubation, while ICU mortality was 40% [104].

## 7. Discussion

Given the beneficial effects of PP in intubated and mechanically ventilated patients with ARDS, the use of awake proning was initiated early during the COVID-19 pandemic [88], with a large randomized meta-trial and several observational studies showing that PP in awake hypoxemic patients with COVID-19 was associated with a reduced need for endotracheal intubation and possibly lower mortality [97,110]. However, although APP was first reported in 1977 [102], evidence supporting its use in spontaneously breathing patients with non-COVID-19 respiratory failure remains limited [104]. Moreover, its physiological effects in non-intubated patients are not fully understood, as most available physiological studies have been conducted in patients with COVID-19 [111,112].

The beneficial effects of APP may be related to a shift in TV from ventral to dorsal lung regions (i.e., from non-dependent to dependent areas), along with improved V/Q matching and pulmonary blood flow patterns [15,106,113]. These physiological mechanisms are well established in patients receiving invasive mechanical ventilation, as PP has been shown to improve gas exchange and reduce LI by decreasing the heterogeneity of mechanical forces. Indeed, reductions in differences in P_PL_ between non-dependent and dependent lung regions are associated with a more homogeneous distribution of ventilation and reopening of atelectatic or insufficiently aerated areas [71,114,115]. Physiological studies in non-intubated patients with non-COVID-19 respiratory failure using EIT to assess the center of ventilation (CoV) and the global inhomogeneity (GI) index have demonstrated a significant increase in CoV and a redistribution of ventilation from ventral to dorsal regions after proning, without changes in TV, lung compliance, driving pressure, or pendelluft magnitude (Table 2) [104,105]. However, despite limited evidence in non-COVID-19 respiratory failure, studies conducted in spontaneously breathing, non-intubated patients with COVID-19 have failed to demonstrate an association between APP and improved ventilation distribution [111,112,116], potentially due to differences in ventilatory support modalities among studies. Another physiological explanation for the improved oxygenation related to the supine-to-prone transition is an increase in end-expiratory lung impedance (EELI), resulting from elevated PEEP and higher transpulmonary end-expiratory pressure [104,117].

Improvements in V/Q mismatch through optimization of pulmonary blood flow may also contribute to enhanced oxygenation [111]. Indeed, from a physiological perspective, shunt and dead space are well recognized as major contributors to V/Q mismatch, significantly affecting gas exchange and oxygenation [114,118]. Clinical studies using EIT in mechanically ventilated patients with ARDS have demonstrated that PP improves the Matched Region%, with a significant decrease in Shunt-EIT%, resulting in improved oxygenation [115]. Similar findings have been reported in non-intubated patients with COVID-19-related respiratory failure, in whom APP improved V/Q matching, reduced dead space fraction, and enhanced oxygenation; however, these beneficial effects were transient and were not maintained after the transition back to the supine position (Table 2) [111]. Recently, Chao et al. [106] showed that APP increased V/Q matching in non-intubated patients with AHRF, including 10 patients with non-COVID-19 respiratory failure. A significant reduction in shunt was observed in patients with diffuse ARDS, whereas no differences were found in those with focal ARDS. Conversely, reductions in dead space were not observed in diffuse ARDS, while patients with focal ARDS experienced a significant decrease in dead space following PP [106]. These findings highlight the importance of ARDS subphenotyping when evaluating the physiological effects of APP [4].

A major concern in the management of spontaneously breathing, non-intubated patients with severe LI or ARDS receiving noninvasive ventilatory support to avoid endotracheal intubation and invasive mechanical ventilation is delayed intubation, which has been associated with increased mortality, as well as the potentially harmful effects of excessive respiratory effort that may contribute to the development of P-SILI [119].

The difference between alveolar and P_PL_, defined as transpulmonary pressure, represents the mechanical “stress” acting on the lungs, leading to their mechanical deformation (strain), and is a major contributor to P-SILI, as it reflects the pressure to which the alveoli are exposed [120,121,122]. In contrast to alveolar pressure, which under static conditions at end-inspiration and end-expiration equals airway pressure, P_PL_ is determined indirectly, based on the anatomical relationship between the pleural cavity and the esophagus, such that P_ES_ is used as a surrogate for P_PL_ [121,123]. Moreover, a strong correlation between the work of breathing and diaphragmatic energy expenditure is well established. These parameters can be assessed at the bedside using esophageal and gastric pressure measurements. Variations in P_ES_ reflect changes in inspiratory effort, while the work of breathing can be monitored using the P_ES_–time product. Accurate quantification, however, requires calculation of chest wall pressure variations [82]. Experimental and clinical studies in individuals without LI as well as in patients with ARDS have shown that a positional change from supine to prone results in an increase in end-expiratory transpulmonary pressure and end-expiratory lung volume, accompanied by a decrease in end-expiratory P_ES_. These changes are associated with increased chest wall elastance and driving pressure due to altered chest wall mechanics [35,36,37,84,124]. Available evidence regarding the effects of PP in non-intubated patients with non-COVID-19-associated respiratory failure is extremely limited. One study, including seven non-COVID-19 patients with respiratory failure and a PaO_2_/FiO_2_ ratio < 200 mmHg receiving HFNC, compared 1 h in the supine position with 2 h in the PP and reported that PP increased ΔP_ES_, likely due to deposition-related increases in airway resistance and prolonged expiratory time [104]. As ΔP_ES_ is a key pathophysiological component in the development of P-SILI, monitoring P_ES_ may significantly contribute to the early identification of patients with vigorous spontaneous breathing efforts and, consequently, those at risk of exacerbation of LI, potentially improving patient prognosis [125,126].

Although the majority of published studies demonstrate an improvement in oxygenation following positional change, when managing non-intubated patients with LI/ARDS using PP, it is important to consider the potential worsening of oxygenation upon returning to the supine position, even if oxygenation initially improves during proning. This phenomenon is not fully understood and may contribute to delays in endotracheal intubation and initiation of invasive mechanical ventilation, potentially resulting in a poorer prognosis [104,127,128]. The severity of hypoxemia remains a major determinant of clinical outcomes [5,104]. In a study by Ding et al. [5], which included 20 patients with AHRF (PaO_2_/FiO_2_ < 200 mmHg) undergoing awake PP, nine patients required endotracheal intubation despite improvements in oxygenation following proning. Among these patients, 22% had moderate ARDS and 78% had severe ARDS, highlighting the importance of considering ARDS severity in clinical decision-making [5]. Indeed, it is well established that patients with moderate to severe ARDS have greater lung weight, exhibit increased lung inhomogeneity and recruitability, and present with more extensive atelectatic regions compared with patients with mild ARDS [129]. Experimental and clinical studies in mechanically ventilated patients with ARDS highlight the potential benefits of spontaneous breathing in mild to moderate ARDS, while recognizing that in severe ARDS, spontaneous breathing may contribute to progression of LI depending on the vigor of respiratory effort and the underlying ARDS etiology [130,131,132]. Finally, early initiation of PP in the course of LI/ARDS and longer durations of proning may be decisive for clinical outcomes and prognosis [5,104].

Although current evidence is insufficient to support the routine implementation of APP in all patients with non-COVID-19 AHRF and/or ARDS, its favorable safety profile and the recent conditional recommendation from the Surviving Sepsis Campaign guidelines (2026) [133] suggest that APP may be considered as an adjunctive intervention to respiratory support in selected non-intubated patients with AHRF. The frequency and duration of APP sessions should be individualized according to patient tolerance. However, close monitoring is essential to avoid delayed escalation of respiratory support, including endotracheal intubation and mechanical ventilation.

## 8. Limitations and Areas of Future Research

Despite its strengths, our study has several limitations. First, the majority of available data regarding the use of PP in spontaneously breathing patients originates from studies performed in mechanically ventilated patients [134]. Moreover, studies in awake, non-intubated individuals predominantly involve patients with COVID-19-associated ARDS. However, it is well established that COVID-19 ARDS differs from typical non-COVID-related ARDS, as it is characterized, beyond the excessive alveolar damage, by distinct pathophysiological features, including endothelial injury, extensive microthrombus formation, and capillary hyperplasia within the lungs [135]. Moreover, the existing literature should be interpreted cautiously, as most studies are based on small patient cohorts, increasing vulnerability to random variability and potentially explaining inconsistent findings. Indeed, given that the available evidence is based primarily on small observational studies, case series, and case reports, the overall certainty of evidence remains low. Confidence in the reported benefits is limited by potential sources of bias, including selection bias, confounding by concurrent treatments, and reporting bias. In addition, marked differences exist among studies with regard to ventilatory support strategies, including HFNC and NIV, as well as study design and methodological approach. Clinical endpoints are heterogeneous, which limits the ability to establish a clear association between APP and patient outcomes. Importantly, randomized controlled trials in this patient population are currently lacking, and existing prospective studies include heterogeneous ARDS phenotypes. Furthermore, the predominance of favorable results in published reports raises concern for publication bias, as neutral or adverse outcomes may be underreported. Finally, despite a generally favorable safety profile, the risk of procedure-related complications should also be considered.

Overall, these limitations highlight the critical need for well-designed, adequately powered, and protocol-standardized randomized clinical trials to elucidate the therapeutic potential of APP in AHRF and ARDS. Important research questions that require further investigation include the optimal timing of APP initiation, the appropriate duration and frequency of sessions, differences among respiratory support strategies, effects on oxygenation, and safety outcomes. Future studies should also focus on long-term outcomes and prognosis. Furthermore, the role of dynamic assessment of ventilation during APP using EIT, a bedside, radiation-free imaging technique that provides continuous real-time monitoring of regional lung ventilation, warrants further investigation [136,137]. Given the existence of distinct subphenotypes within AHRF and ARDS, future research should evaluate the effects of APP in specific patient subgroups. In addition, the identification of biomarkers capable of recognizing patients most likely to benefit from APP and elucidating the mechanisms underlying response to APP may significantly contribute to understanding the pathophysiology and improving the management of this vulnerable population [138].

## 9. Conclusions

Early application of PP in spontaneously breathing, non-intubated patients with moderate non-COVID-19 AHRF and ARDS, in combination with HFNC and/or NIV, may improve gas exchange by redistributing TV from ventral to dorsal lung regions, reducing lung inhomogeneity, and decreasing respiratory effort. However, worsening of oxygenation upon transition from prone to supine position, the potential development of P-SILI, and delays in endotracheal intubation and initiation of invasive mechanical ventilation with possible adverse effects on prognosis remain major concerns. Well-designed experimental studies and multicenter prospective randomized controlled trials in non-intubated patients with non-COVID-19 ARDS are needed to further clarify its therapeutic role, particularly in light of its generally favorable safety profile. Until data from randomized controlled trials become available, the use of APP in patients with AHRF and ARDS should be individualized, considered in carefully selected patients, and implemented with close monitoring.
**Questions for Future Research**How does APP influence lung recruitment when combined with different forms of respiratory support such as HFNC or NIV?What impact does APP have on patient respiratory effort and the risk of P-SILI?Can randomized controlled trials confirm whether awake prone positioning reduces intubation rates in non-COVID-19-related AHRF?Does APP modulate pulmonary and systemic inflammatory responses in AHRF and ARDS?

## Figures and Tables

**Table 1:** Summary of studies evaluating awake prone positioning in non-intubated patients with non-COVID-19 acute hypoxemic respiratory failure and ARDS.

Study	Study Design	Patients (n)	Ventilatory Support	Duration of APP	Main Findings
**Valter et al.** [22]	Case series	4	NIV, CPAP	1–5 h	Rapid improvement of oxygenation; intubation avoided in 4/4 patients
**Feltracco et al.** [87]	Case series	2	NIV	6–8 h, several days	Improved oxygenation; intubation avoided in 3/3 patients
**Feltracco et al.** [101]	Case series	3	HFPV	1–3 h, 3–6 times daily	Improved oxygenation; decreased respirator effort
**Scaravilli et al.** [23]	Retrospective study	15	HFNC, NIV, CPAP	Median duration 3 h daily, 2 days	Improved oxygenation; ICU mortality 20%; intubation avoided in 13/15 patients
**Ding et al.** [5]	Prospective cohort study	20	HFNC, NIV	30 min twice daily, ≥3 days	Improved oxygenation; intubation avoided in 11/20; intubation in 67% of patients with severe ARDS
**Wang et al.** [103]	Case report	1	HFNC	Continuous	Improvement of oxygenation
**Pérez-Nieto et al.** [24]	Retrospective case series	6	HFNC, NIV	2–3 h twice daily, 2 days	Improved oxygenation; intubation avoided in 4/6 patients
**Grieco et al.** [104]	Sequential crossover study	7	HFNC	2 h	Improved oxygenation, reduced respiratory rate, improved VT distribution, increased Δ*P*_ES_
**Wang et al.** [105]	Prospective physiological study	10	HFNC	Average duration 2 h (IQR: 2–4.5)	Significant increase in ROX, improvement in ventilation distribution, and homogeneity of lung ventilation
**Chao et al.** [106]	Prospective physiological study	10	Nasal tube oxygen, HFNC	2 h	Improved oxygenation, V/Q matching, shunt reduction in pt with diffuse opacities
**Pacchiarini et al.** [107]	Case report	1	Mask	Two cycles of 4 and 8 h	Improvement of gas exchange and respiratory function
**Choudhuri et al.** [108]	Case report	1	HFNC, NIV	≥8 h/day, prolonged	Improvement of oxygenation
**Jiang et al.** [109]	Case report	1	HFNC	≥12 h/day	Improvement of oxygenation

ARDS: acute respiratory distress syndrome; CPAP: continuous positive airway pressure; Δ*P*_ES_: inspiratory effort; HFNC: high-flow nasal cannula; HFPV: high-frequency percussive ventilation; ICU: intensive care unit; NIV: non-invasive ventilation; V/Q: ventilation–perfusion.

**Table 2:** Summary of pulmonary and hemodynamic effects of awake prone positioning in acute hypoxemic respiratory failure.

Physiological Effect of APP	Reported Findings	References
**Oxygenation**	Increased PaO_2_/FiO_2_ ratio and improved oxygenation during APP	[5,22,23,24,87,103,106,107,108,109]
**Ventilation distribution**	Improved ventilation distribution with redistribution of tidal ventilation toward dorsal lung regions without changes in overall tidal volume	[104,105]
**Ventilation homogeneity**	Increased ventilatory homogeneity assessed by EIT parameters	[105]
**Ventilation–perfusion matching**	Improved V/Q matching with potential reduction in intrapulmonary shunt and dead space	[106]
**Respiratory pattern**	Reduced respiratory rate and changes in inspiratory effort during APP	[104,107]
**Non-invasive respiratory support outcomes**	Combination of HFNC or NIV with APP associated with avoidance of endotracheal intubation in selected patients	[5,23,24,87]
**Hemodynamic effects**	No significant hemodynamic alterations	[23]
**Overall physiological response**	Increase in ROX index, suggesting improved oxygenation and respiratory status	[105]

APP: awake prone positioning; EIT: electrical impedance tomography; HFNC: high-flow nasal cannula; NIV: non-invasive ventilation; V/Q: ventilation–perfusion.

## Data Availability

No new data were created or analyzed in this study. Data sharing is not applicable to this article.
